# Normative emotional responses to environmental sounds in middle-aged to older adults with normal hearing: Stimuli from the Marcell database

**DOI:** 10.1371/journal.pone.0356295

**Published:** 2026-08-21

**Authors:** Dina Lelic, Frederic Marmel

**Affiliations:** ORCA Labs, Lynge, Denmark; Dresden University of Technology, GERMANY

## Abstract

**Purpose:**

This study aimed to provide normative affective ratings for middle-aged to older adults with normal hearing using the Marcell database. The database includes 120 diverse environmental sounds representing various real-world acoustic events, such as those produced by animals, humans, musical instruments, tools, signals, and liquids. Secondary aims were to examine the relationship between valence and arousal, and whether factors such as age, gender, hearing-related lifestyle, hearing demands, and self-reported hearing difficulties influence these affective evaluations.

**Study sample:**

Ninety participants (mean age: 54 ± 9 years) with self-reported normal hearing.

**Data collection and analysis:**

The study was conducted online. Participants rated each sound token in the Marcell database in terms of valence (pleasantness) and arousal (intensity). They also completed a hearing-related lifestyle questionnaire assessing the frequency, importance, and difficulty of hearing in everyday situations. Normative data for valence and arousal were calculated as means and standard deviations across participants for each individual sound token. To explore the relationship between valence and arousal, correlation analyses were performed. Furthermore, mixed-effects linear regression was used to examine whether any of the measured variables could predict participants’ affective responses to the sounds.

**Results:**

Normative data for each individual sound token were obtained and reported. A moderately strong negative correlation was found between valence and arousal ratings, indicating that pleasant sounds were generally perceived as calming, while unpleasant sounds were seen as more arousing. Regression analyses revealed that age, gender, hearing-related lifestyle, hearing demands, and self-reported hearing difficulties did not significantly predict valence or arousal ratings in the current dataset of middle-aged to older adults.

**Conclusions:**

The normative data presented in this study offer a benchmark for the hearing research community when examining emotional responses across middle-aged to older adult populations.

## Introduction

Environmental sounds play a fundamental role in everyday auditory experiences, serving not only as cues for situational awareness but also as carriers of emotional information [1–3]. The affective perception of these sounds is increasingly recognized as relevant to audiological research, particularly in understanding how hearing loss and hearing assistive devices influence the affective perception [4–8], and how these affective perceptions are related to well-being [9]

Affective sound perception is commonly described along two primary dimensions: valence, which reflects the pleasantness of a sound, and arousal, which indicates the intensity or activation level it elicits [10]. One database that has been designed for affective research along these two dimensions is the International Affective Digitized Sounds corpus (IADS) [11]. The IADS consists of 167 environmental non-speech sounds of 6-second length, which vary along the valence and arousal dimensions. The IADS has been used in a number of hearing research studies which showed that valence ratings were lower for people with hearing loss than their peers with normal hearing [5,6], people with hearing loss were slower to respond to affective stimuli in comparison to those with normal hearing [8], people with hearing loss and users of hearing assistive devices had a reduced range of valence ratings [4,5], and emotional responses to pleasant sounds were related to social disconnectedness and loneliness [9]. Although the IADS has yielded valuable insights into the relationship between emotional responses to sounds, hearing loss, and well-being, it does come with certain limitations. The database comprises 167 stimuli, all standardized to the same duration. This uniformity can be problematic. For instance, assigning the same length to a brief, transient sound like glass breaking and a prolonged, continuous sound like howling wind may not accurately reflect their natural characteristics. Additionally, the total number of stimuli may be too large for inclusion in a single study without risking participant fatigue. As a result, many studies opt to use only a subset of the stimuli [4–6,8,9], and in some studies the length is reduced to 1.5 seconds [4–6,9]. This selective use can hinder cross-study comparisons unless all researchers consistently use the same subset and apply identical processing methods.

A more recent database titled Norms for Environmental Sound Stimuli (NESSTI) was developed [12]. The database consists of 110 environmental sounds, including living (e.g., animal) and manmade (e.g., machinery) sounds normalized to 1-second length. The authors provide normative valence and arousal data for younger listeners. However, to our knowledge, NESSTI has not yet been applied in audiological affective research. Like IADS, NESSTI also shares the limitation of using sound tokens with uniform durations. Additionally, a large proportion of sound tokens in the NESSTI database fall into the neutral category [12], which may limit their suitability for studies aiming to elicit emotional responses. The authors attribute this predominance of neutral ratings to the brevity of the sound clips, highlighting another limitation of the database for affective research in the auditory domain.

Marcell and collaborators [13] developed a database comprising 120 naturalistic non-speech sounds of varying durations in respect to sound source and event, representing a broad spectrum of real-world acoustic events. These sounds include those produced by animals, humans, tools, musical instruments, and warning signals. The Marcell database emphasizes ecological validity by editing each sound to a duration believed to allow the sound event or auditory object to unfold naturally. Furthermore, the Marcell database is freely available to researchers and clinicians, facilitating reproducibility and cross-study comparisons in both basic and applied auditory research. In terms of affective ratings, Marcell and collaborators [13] report normative data for valence, but not arousal, for younger individuals (college students) with normal hearing, and the authors report a range of sounds that fall into positive valence category as well as negative. The variability in environmental sounds and their length, the range of valence responses, and the availability of the Marcell database make it very useful for hearing research. However, given that middle-aged to older adults represent the primary population affected by hearing loss [14], it is important to establish normative affective responses in this population to eventually better understand how such responses are influenced by hearing loss or use of hearing assistive devices. Defining normative affective responses can benefit the hearing research community in two key ways: 1) by offering categorized examples of pleasant, neutral, and unpleasant sounds as perceived by middle-aged to older adults, which can support analyses involving this demographic; and 2) by establishing a baseline of valence and arousal ratings that can serve as a reference point for comparisons with other subgroups of middle-aged to older adults.

The primary aim of the present study was to establish normative valence and arousal ratings for the environmental sounds originally compiled by Marcell and collaborators [13], specifically in a population of middle-aged to older adults who self-reported normal hearing. Secondary objectives were to a) assess whether there is a relationship between valence and arousal ratings, and b) explore whether individual characteristics – such as age, gender, or self-reported situational hearing difficulties – could predict variations in emotional responses to these sounds. Prior research has demonstrated that both aging and hearing loss can influence emotional reactions to auditory stimuli [5,7,15]. Although participants in this study reported normal hearing, it is important to recognize that individuals with clinically normal hearing may still encounter varying degrees of hearing difficulties depending on the situation.

## Materials and methods

Ethical waiver for conducting the study was given by the Research Ethics Committee of the Capital Region of Denmark (case no. H-18056647). All the participants were informed about the purpose of the study and how their data would be handled in writing, and they gave their written consent to participate before entering the study.

The experiment was created with PsychoPy [16], hosted on Pavlovia (https://pavlovia.org/), and conducted online. The Prolific platform (www.prolific.com) was used to recruit the participants and direct them to the online experiment. The Prolific platform provides demographic data on the participants.

### Participants

The inclusion criteria were: self-reported normal hearing, 40–80 years of age, and fluency in English. Exclusion criteria were: answering the attention check questions wrong and completing the study too quickly, i.e., in less than 25 minutes, as this could indicate careless responses. Hundred-forty-four participants were recruited between January 17 – February 5, 2025. Of the recruited participants, 32 were excluded because they experienced technical issues with the platform which hindered them from successfully completing the study. Further 22 participants were excluded because they failed attention checks. This resulted in 90 participants successfully completing the study (age: 54 ± 9, gender: 54 females, 36 males).

### The experimental flow

Participants were instructed to use headphones for the listening part of the experiment.

**Fig 1:**
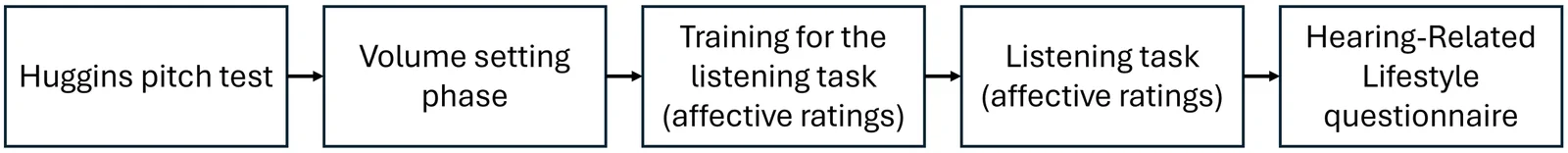
The experimental flow.

They were also instructed that if they did not have headphones they could use earbuds, but headphones were preferred. The experimental flow is depicted in Fig 1.

Participants completed the dichotic Huggins pitch (HP) test proposed by Milne and collaborators to verify headphone or earbud usage [17]. HP is a dichotic pitch generated by presenting white noise in both ears, but with a narrow frequency band being phase-shifted by 180º between ears. The binaural processing of these two different signals creates a faint pitch percept at the center of the phase-shifted frequency band. While we did not use the HP test as a screening tool, it served as a post hoc quality check. The test has been shown to correctly identify approximately 80% of headphone users [17]. Accordingly, we verified that our pass rate was close to this benchmark and compared the results with and without excluding participants who failed the test to ensure consistency before analyzing the full dataset. We chose not to exclude participants based on HP test performance because prior research indicates that such exclusions have minimal if any impact on perceptual data outcomes [18]. Moreover, exclusion based on headphone checks can introduce demographic biases, such as disparities in age, gender, education, and geographic location, which may have more significant implications for the study’s generalizability [18].

After the HP test, participants set the volume on their computer for the remainder of the experiment: a series of three sound tokens from the Marcell database covering the full range of sound levels of the experimental stimuli set was played in loop, and participants were asked to adjust their computer volume until comfortable for all three sound tokens.

Participants then received instructions for the listening task. They were informed that they would be rating a series of sounds based on their emotional responses. Specifically, they would evaluate each sound along two dimensions: valence (how pleasant or unpleasant the sound made them feel) and arousal (how calming or exciting the sound was perceived to be). To facilitate these ratings, the Self-Assessment Manikin (SAM) scale was used [19]. For each dimension, the SAM includes five figures, each representing an emotion that varies along that specific dimension. The valence scale appeared at the top, ranging from a very unhappy to a very happy figure, while the arousal scale was shown below, ranging from a very calm to a very excited figure. Each dimension includes a 9-point Likert scale, ranging from 1 = low to 9 = high, with the two end points visually aligned with the center of the two extreme figures. Higher scores indicate higher ratings of arousal (more exciting) and higher ratings of valence (more pleasant). The concepts of valence and arousal were explained with examples. For valence, participants were told that, if a sound like birdsong was perceived as very pleasant, they should select the figure on the right; if a loud siren was perceived as very unpleasant, they should choose the figure on the left. For arousal, they were told that a gentle breeze might be rated as calming (left), whereas a loud explosion might be rated as highly exciting (right). They were also instructed that valence and arousal ratings are independent. For instance, a sound high in arousal could be either pleasant (e.g., laughter, fast music) or unpleasant (e.g., a child crying, a gunshot).

Auditory stimuli were tokens from the Marcell database. The database consists of 120 sounds that vary along the valence and arousal dimensions. These sounds range in duration from brief, transient events like a cork popping (137 milliseconds) to extended recordings such as a helicopter (5.937 seconds), preserving the natural temporal characteristics of each sound. Participants rated all 120 sounds, presented in a random order (without replacement). Prior to the main listening task, participants completed a brief familiarization phase in which they rated three practice sounds (taken from an internally available environmental sound database) on both emotional dimensions. Following this, they proceeded to evaluate all 120 sound clips. A one-minute break was enforced after every 30 sounds. Participants had the possibility to skip a sound if they did not hear it, in which case they selected “I did not hear the sound.”

After completing the listening task, participants filled out the Hearing-Related Lifestyle Questionnaire (HEARLI-Q; Lelic et al. [20]). This questionnaire assesses hearing experiences across 23 everyday listening situations, which participants rate on three dimensions: how often the situation occurs, how important it is to hear well in that context, and how difficult it is to hear. From these responses, three composite scores are derived: richness of hearing-related lifestyle and hearing demand (both ranging from 0 to 100) and hearing difficulty (ranging from 0 to 4). Higher scores reflect greater lifestyle richness, higher hearing demands, and increased difficulty. The HEARLI-Q has shown excellent short-term reliability and good-to-excellent reliability over longer periods [20].

This study formed part of a broader data collection effort that included four additional questionnaires alongside the HEARLI-Q. To ensure participant attentiveness, three attention checks were embedded within the questionnaires. Participants who failed any of the attention checks were excluded from the analyses.

### Data analysis

The normative data are reported as the mean and standard deviation (SD) of individual sound tokens for both valence and arousal ratings. To assess consistency with previous findings in younger individuals, the calculated valence scores from the current study were correlated with those reported by Marcell and collaborators [13] using Spearman correlation. Additionally, Spearman correlation was used to examine the relationship between valence and arousal scores within the current dataset.

To explore potential predictors of valence and arousal, two mixed-effects linear regression models were conducted. Predictor variables included age, gender, hearing-related lifestyle, hearing demand, and self-reported hearing difficulty. Valence and arousal ratings of individual sound tokens were the outcome variables. In both models, participant ID and sound token ID were included as random intercepts. Residuals were visually inspected to verify approximate normality and homoscedasticity.

All analyses were performed using Stata version 15 (StataCorp, College Station, TX, USA).

## Results

Out of 90 participants, 72 (80%) passed the HP test. We compared the normative data both with and without the 18 participants who did not pass the test. The results were similar across both datasets in terms of average valence and arousal scores. Excluding participants who failed the HP test would not have affected the categorization of tokens into pleasant, unpleasant, high arousal, or low arousal categories. As explained in the Methods, we chose to retain all participants in the analysis.

Due to technical problems with the online sound playback, there are some missing data. Fourteen participants reported at least one sound token not being presented. This problem did not affect any one sound specifically. One participant reported missing five tokens, whereas the remaining participants missed only one token.

### Valence and arousal normative data

**Fig 2:**
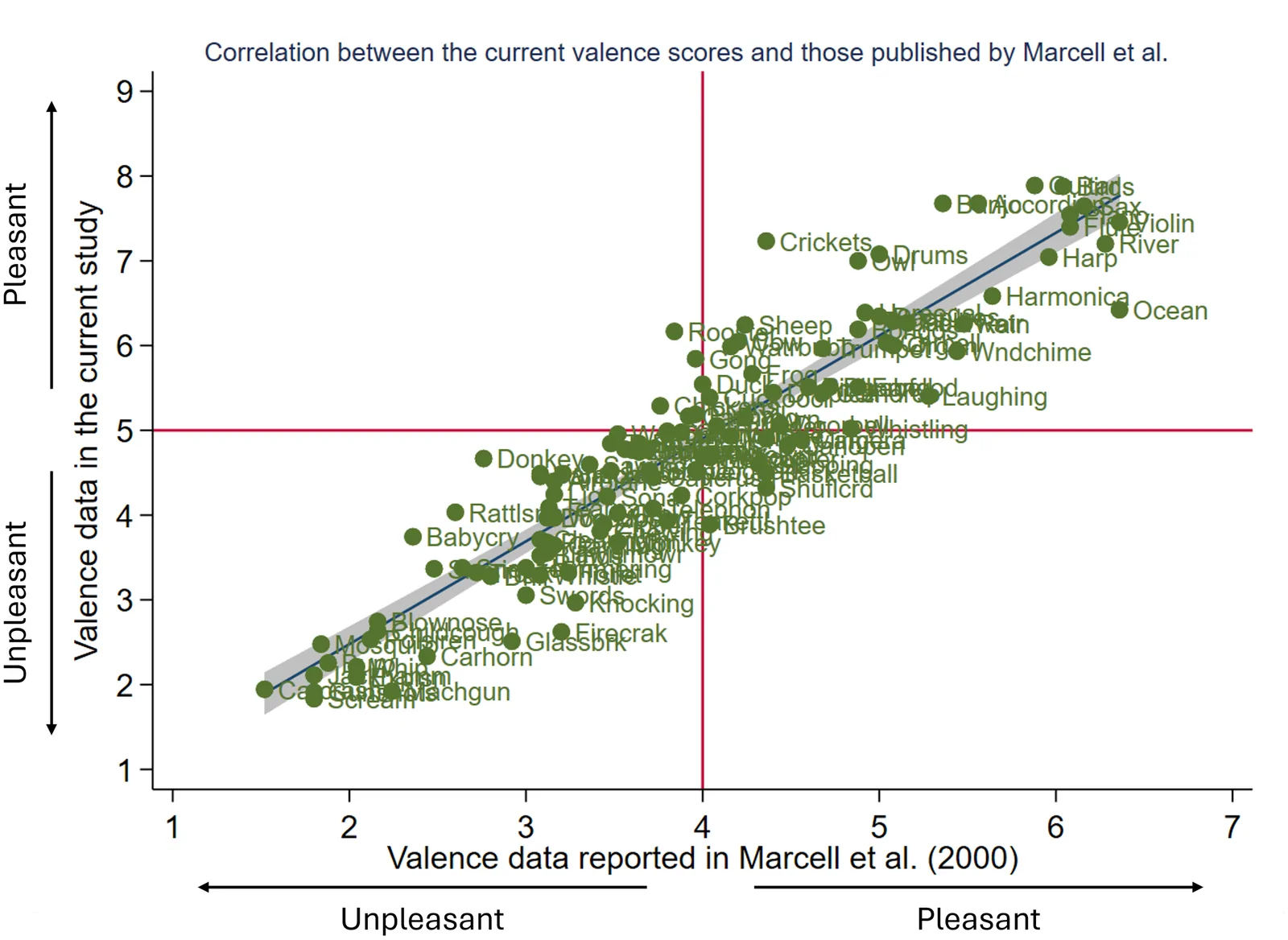
Scatter plot and line of best fit of valence scores for individual sound tokens in the current study versus those reported in Marcell et al. [13]. The gray area around the line of best fit represents the 95% confidence interval around the line. The horizontal line depicts the neutral score in the current study (5 on a 9-point scale) and the vertical line depicts the neutral score in the Marcell et al. [13] study (4 on a 7-point scale).

The mean and SD of valence and arousal ratings for individual sound tokens are presented in Table 1. Valence scores ranged between 1.83–7.89. The five most pleasant sounds were guitar, birds chirping, accordion, banjo, and saxophone, whereas the five least pleasant sounds were scream, gunshots, machine gun, car crash, and explosion (see Table 2). Valence scores showed a strong positive correlation with those reported by Marcell and collaborators [13] in younger individuals (ρ = 0.91, p < .0001; see Fig 2). Arousal scores ranged between 3.34–7.84. The five most calming sounds were yawning, birds chirping, crickets, river, and pouring water, whereas the five most arousing sounds were machine gun, gunshots, scream, explosion, and car crash (see Table 3). Marcell and collaborators [13] did not report arousal ratings in their work, hence, we could not correlate our findings with theirs.

**Table 1:** Mean valence and arousal ratings for individual sound tokens.

Sound Label	Valence Average (SD)	Arousal Average (SD)
Accordion	7.68 (1.56)	4.00 (2.41)
Airplane	4.40 (2.16)	5.92 (1.94)
Baby crying	3.74 (2.06)	6.59 (1.77)
Bagpipes	6.34 (2.10)	6.04 (1.93)
Banjo	7.68 (1.18)	5.49 (2.16)
Basketball	4.50 (1.89)	5.43 (1.61)
Bicycle bell	5.51 (1.65)	5.32 (1.61)
Birds chirping	7.88 (1.34)	3.44 (2.29)
Blinds	3.52 (1.56)	5.37 (1.74)
Blowing nose	2.74 (1.29)	5.21 (1.74)
Boat horn	4.84 (1.88)	5.33 (1.76)
Bongos	6.19 (1.70)	4.88 (1.89)
Bowling	4.90 (1.97)	5.87 (1.89)
Brushing teeth	3.89 (1.75)	4.98 (1.69)
Burp	2.26 (1.55)	5.60 (2.04)
Camera	4.89 (1.50)	5.23 (1.77)
Can crush	4.44 (1.27)	4.64 (1.70)
Can opening	4.82 (1.29)	4.71 (1.67)
Car crash	1.94 (1.21)	7.17 (1.96)
Car horn	2.33 (1.31)	6.94 (1.89)
Cash register	5.17 (1.39)	5.16 (1.75)
Cat	6.29 (1.98)	4.70 (1.98)
Chewing	3.81 (1.85)	5.00 (1.94)
Chickens	5.29 (1.80)	5.34 (1.70)
Child coughing	2.63 (1.42)	5.96 (1.78)
Church bells	6.03 (2.05)	5.09 (2.14)
Clapping	4.60 (1.83)	5.80 (1.68)
Clearing throat	3.71 (1.29)	5.10 (1.69)
Coin dropping	5.44 (1.19)	4.64 (1.67)
Cork popping	4.23 (1.67)	5.53 (1.75)
Cow	6.04 (1.73)	4.27 (1.74)
Crickets	7.23 (1.61)	3.48 (2.03)
Crow	4.02 (2.03)	5.68 (1.83)
Crumpling paper	4.74 (1.47)	4.39 (1.64)
Cuckoo clock	5.39 (2.08)	5.32 (1.85)
Cutting paper	4.78 (1.22)	4.37 (1.54)
Cymbals	4.98 (1.72)	5.71 (1.78)
Dog barking	4.53 (2.07)	5.62 (1.95)
Donkey	4.67 (2.15)	5.58 (1.70)
Doorbell	5.07 (1.77)	5.59 (1.82)
Door closing	3.97 (1.28)	5.22 (1.57)
Drill	3.28 (1.48)	5.48 (1.82)
Dropping ice in glass	5.44 (1.50)	4.41 (1.68)
Drums	7.08 (1.45)	5.88 (1.94)
Duck	5.54 (1.91)	5.46 (1.74)
Elephant	4.77 (2.24)	6.22 (1.80)
Explosion	2.09 (1.69)	7.37 (1.83)
Firecrackers	2.62 (1.65)	6.91 (1.73)
Flute	7.40 (1.79)	3.93 (2.43)
Frog	5.67 (1.79)	4.40 (1.69)
Frying food	5.51 (1.76)	4.42 (1.63)
Gargling	3.64 (1.74)	4.96 (1.68)
Glass breaking	2.51 (1.25)	6.56 (1.77)
Gong	5.84 (1.75)	4.90 (1.99)
Guitar	7.89 (1.43)	3.60 (2.24)
Gunshots	1.91 (1.40)	7.66 (1.64)
Hammering	3.38 (1.39)	5.90 (1.65)
Harmonica	6.58 (2.01)	5.40 (2.20)
Harp	7.04 (1.61)	4.62 (2.04)
Helicopter	4.68 (1.90)	5.56 (1.81)
Horse galloping	6.39 (1.44)	5.29 (1.91)
Jackhammer	2.11 (1.44)	6.96 (1.64)
Knocking	2.97 (1.51)	6.63 (1.86)
Laughing	5.40 (2.16)	5.74 (1.50)
Lawn mower	3.56 (1.71)	5.86 (1.63)
Lion	4.24 (2.05)	6.60 (1.92)
Machine gun	1.92 (1.33)	7.84 (1.62)
Monkey	3.68 (1.83)	6.16 (1.67)
Mosquito	2.48 (1.57)	6.77 (1.73)
Motorcycle	4.52 (2.07)	5.57 (1.62)
Ocean	6.42 (2.13)	4.10 (2.45)
Organ	6.00 (1.92)	5.16 (1.87)
Owl	7.00 (1.70)	3.60 (1.99)
Piano	7.54 (1.30)	5.48 (2.16)
Pig	3.68 (1.84)	5.50 (1.67)
Pinball	5.19 (1.37)	4.93 (1.74)
Ping-pong	5.51 (1.64)	4.72 (1.93)
Police siren	2.53 (1.53)	6.80 (1.95)
Pouring water	6.27 (1.49)	3.58 (1.61)
Pullchain lightswitch	4.78 (1.26)	4.48 (1.69)
Rain	6.26 (2.14)	3.72 (2.00)
Rattlesnake	4.03 (1.62)	5.34 (1.62)
River	7.20 (1.69)	3.49 (2.25)
Rooster	6.17 (1.90)	4.96 (1.87)
Sandpaper	4.49 (1.51)	4.79 (1.87)
Sawing	4.60 (1.59)	5.00 (1.64)
Saxophone	7.64 (1.48)	4.30 (2.55)
Scream	1.83 (1.10)	7.62 (1.70)
Seal	4.99 (1.83)	5.14 (1.79)
Sheep	6.24 (1.74)	4.52 (2.02)
Shuffling cards	4.32 (1.75)	4.91 (1.76)
Sneeze	3.38 (1.66)	5.07 (1.83)
Snoring	3.37 (1.74)	5.10 (1.99)
Sonar	4.22 (1.73)	5.06 (1.74)
Stapler	4.52 (1.39)	4.72 (1.69)
Swords	3.06 (1.30)	6.00 (1.66)
Tea kettle	3.93 (2.03)	5.50 (1.89)
Tearing paper	4.09 (1.35)	4.98 (1.49)
Telephone	4.08 (1.74)	6.36 (1.72)
Thunder	5.06 (2.40)	5.89 (2.17)
Toilet	3.32 (1.48)	4.59 (1.72)
Train	4.84 (1.93)	5.98 (1.90)
Truck	3.32 (1.75)	5.91 (1.68)
Trumpet	5.97 (1.84)	6.09 (1.71)
Turning pages	4.96 (1.39)	4.38 (1.67)
Typewriter (manual)	4.72 (1.48)	5.29 (1.65)
Velcro	4.49 (1.42)	4.68 (1.56)
Violin	7.46 (1.66)	4.00 (2.23)
Water bubbling	5.99 (1.67)	3.69 (1.66)
Water draining	4.70 (1.69)	4.18 (1.74)
Water dripping	4.96 (1.68)	4.23 (1.65)
Whip	2.21 (1.25)	6.83 (1.83)
Whistle (instrument)	3.29 (1.88)	6.58 (1.71)
Whistling (lips)	5.02 (1.95)	5.38 (1.69)
Wind	4.93 (1.91)	4.83 (1.92)
Wind chimes	5.93 (1.92)	4.44 (1.87)
Wolf	4.46 (2.35)	6.28 (1.95)
Woodpecker	3.97 (1.68)	5.47 (1.86)
Yawning	5.14 (1.61)	3.34 (1.58)
Zipper	3.89 (1.58)	5.38 (1.63)

* These data are also shared in a Supplemental Excel file (S1 Table).

**Table 2:** 120 sounds listed in descending order of valence (Most → Least Pleasant).

1. Guitar	31. Frog	61. Typewriter (manual)	91. Clearing throat
2. Birds chirping	32. Duck	62. Water draining	92. Monkey
3. Accordion	33. Bicycle bell	63. Helicopter	93. Pig
4. Banjo	34. Frying food	64. Donkey	94. Gargling
5. Saxophone	35. Ping-pong	65. Clapping	95. Lawn mower
6. Piano	36. Coin dropping	66. Sawing	96. Blinds
7. Violin	37. Dropping ice in glass	67. Dog barking	97. Hammering
8. Flute	38. Laughing	68. Motorcycle	98. Sneeze
9. Crickets	39. Cuckoo clock	69. Stapler	99. Snoring
10. River	40. Chickens	70. Basketball	100. Toilet
11. Drums	41. Pinball	71. Sandpaper	101. Truck
12. Harp	42. Cash register	72. Velcro	102. Whistle (instrument)
13. Owl	43. Yawning	73. Wolf	103. Drill
14. Harmonica	44. Doorbell	74. Can crush	104. Swords
15. Ocean	45. Thunder	75. Airplane	105. Knocking
16. Horse galloping	46. Whistling (lips)	76. Shuffling cards	106. Blowing nose
17. Bagpipes	47. Seal	77. Lion	107. Child coughing
18. Cat	48. Cymbals	78. Cork popping	108. Firecrackers
19. Pouring water	49. Turning pages	79. Sonar	109. Police siren
20. Rain	50. Water dripping	80. Tearing paper	110. Glass breaking
21. Sheep	51. Wind	81. Telephone	111. Mosquito
22. Bongos	52. Bowling	82. Rattlesnake	112. Car horn
23. Rooster	53. Camera	83. Crow	113. Burp
24. Cow	54. Boat horn	84. Door closing	114. Whip
25. Church bells	55. Train	85. Woodpecker	115. Jackhammer
26. Organ	56. Can opening	86. Tea kettle	116. Explosion
27. Water bubbling	57. Cutting paper	87. Brushing teeth	117. Car crash
28. Trumpet	58. Pullchain lightswitch	88. Zipper	118. Machine gun
29. Wind chimes	59. Elephant	89. Chewing	119. Gunshots
30. Gong	60. Crumpling paper	90. Baby crying	120. Scream

**Table 3:** 120 sounds listed in ascending order of arousal (Most Calming → Most Arousing).

1. Yawning	31. Velcro	61. Bicycle bell	91. Drums
2. Birds chirping	32. Cat	62. Cuckoo clock	92. Thunder
3. Crickets	33. Can opening	63. Boat horn	93. Hammering
4. River	34. Ping-pong	64. Chickens	94. Truck
5. Pouring water	35. Stapler	65. Rattlesnake	95. Airplane
6. Guitar	36. Sandpaper	66. Blinds	96. Child coughing
7. Owl	37. Wind	67. Whistling (lips)	97. Train
8. Water bubbling	38. Bongos	68. Zipper	98. Swords
9. Rain	39. Gong	69. Harmonica	99. Bagpipes
10. Flute	40. Shuffling cards	70. Basketball	100. Trumpet
11. Accordion	41. Pinball	71. Duck	101. Monkey
12. Violin	42. Gargling	72. Woodpecker	102. Elephant
13. Ocean	43. Rooster	73. Drill	103. Wolf
14. Water draining	44. Brushing teeth	74. Piano	104. Telephone
15. Water dripping	45. Tearing paper	75. Banjo	105. Glass breaking
16. Cow	46. Chewing	76. Pig	106.Whistle (instrument)
17. Saxophone	47. Sawing	77. Tea kettle	107. Baby crying
18. Cutting paper	48. Sonar	78. Cork popping	108. Lion
19. Turning pages	49. Sneeze	79. Helicopter	109. Knocking
20. Crumpling paper	50. Church bells	80. Motorcycle	110. Mosquito
21. Frog	51. Clearing throat	81. Donkey	111. Police siren
22. Dropping ice in glass	52. Snoring	82. Doorbell	112. Whip
23. Frying food	53. Seal	83. Burp	113. Firecrackers
24. Wind chimes	54. Cash register	84. Dog barking	114. Car horn
25. Pullchain lightswitch	55. Organ	85. Crow	115. Jackhammer
26. Sheep	56. Blowing nose	86. Cymbals	116. Car crash
27. Toilet	57. Door closing	87. Laughing	117. Explosion
28. Harp	58. Camera	88. Clapping	118. Scream
29. Can crush	59. Horse galloping	89. Lawn mower	119. Gunshots
30. Coin dropping	60. Typewriter (manual)	90. Bowling	120. Machine gun

### Relationship between valence and arousal

**Fig 3:**
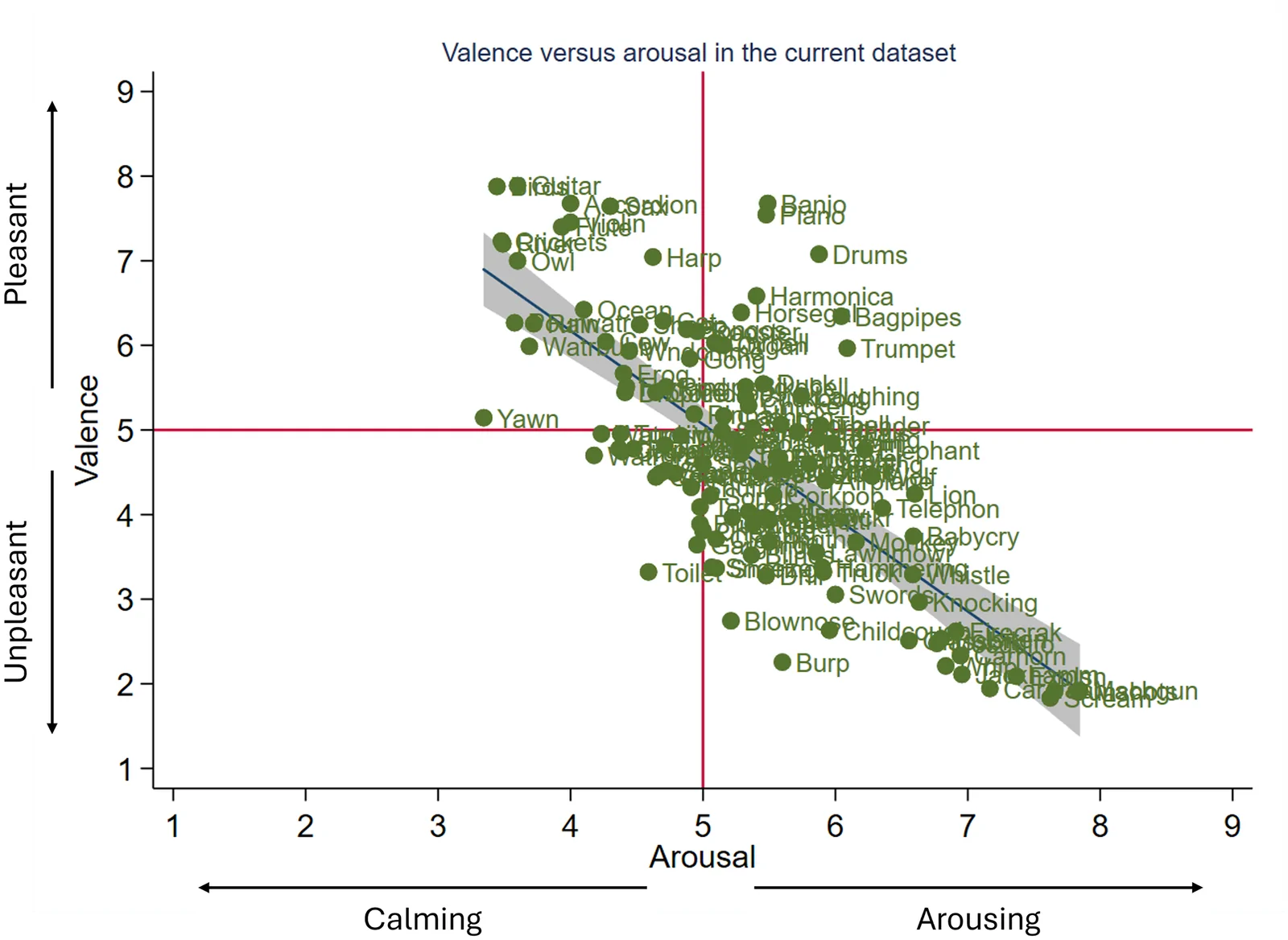
Scatter plot and line of best fit of valence versus arousal scores for individual sound tokens in the current dataset. The gray area around the line of best fit represents the 95% confidence interval around the line. The horizontal line depicts the neutral score for valence and the vertical line depicts the neutral score for arousal.

A moderately strong negative correlation was observed between valence and arousal scores (ρ = 0.64, *p* < .0001; see Fig 3), indicating that pleasant sounds were generally perceived as calming, while unpleasant sounds were typically experienced as arousing. However, closer inspection of Fig 3 reveals a more nuanced pattern: sounds rated as pleasant (high valence) are distributed across a range of arousal levels. In contrast, sounds rated as unpleasant (low valence) show a clearer trend – lower valence ratings seem to be associated with higher arousal. To examine this further, we conducted separate correlation analyses for pleasant sounds (valence ≥ 6) and unpleasant sounds (valence ≤ 4). For pleasant sounds, the correlation was weak and non-significant (ρ = −0.28, *p* = .16), whereas for unpleasant sounds, the correlation was strong and highly significant (ρ = −0.76. *p* < .0001). These findings suggest that while the unpleasant sounds of the Marcell database are linked to high arousal, the pleasant sounds can evoke either calming or arousing responses.

### Predictors of valence and arousal

Valence and arousal were not significantly influenced by age, gender, hearing-related lifestyle, hearing demand, or hearing difficulty in the current dataset (see Table 4 for detailed statistics).

**Table 4:** Results of regression analyses examining whether age, gender, hearing-related lifestyle, hearing demand, or hearing difficulty predict valence or arousal.

Outcome	Predictor	β	95% CI	*p*-value
Valence	Age	−0.00	−0.02, 0.01	.95
	Gender	−0.01	−0.28, 0.26	.96
	Hearing-Related Lifestyle	0.00	−0.01, 0.02	.69
	Hearing demand	−0.01	−0.02, 0.01	.60
	Hearing difficulty	−0.23	−0.54, 0.07	.14
Arousal	Age	−0.01	−0.03, 0.02	.57
	Gender	−0.13	−0.52, 0.26	.52
	Hearing-Related Lifestyle	0.01	−0.02, 0.03	.53
	Hearing demand	−0.02	−0.05, 0.01	.19
	Hearing difficulty	0.40	−0.05, 0.84	.08

## Discussion

In this study, we established normative valence and arousal ratings for environmental sounds from the Marcell database, specifically for middle-aged to older adults who self-reported normal hearing. Our findings revealed an inverse relationship between valence and arousal. Furthermore, individual factors such as age, gender, hearing-related lifestyle, hearing demands, and self-reported hearing difficulties did not significantly affect these ratings in the current sample.

### Normative data

The valence and arousal ratings covered a broad range, indicating that the sounds were not concentrated around neutral values. This wide emotional range makes the Marcell database a robust tool for studying emotional responses to sound. Notably, the valence ratings for middle-aged to older adults strongly aligned with those previously reported for younger adults [13], suggesting a consistency in the pleasantness perception of environmental sounds across different adult age groups with normal hearing. The normative ratings of valence and arousal provided by this study can serve as a reference for the hearing research community when analyzing data from various groups of middle-aged to older adults, such as individuals with hearing loss or those using hearing aids.

### Relationship between valence and arousal

An inverse relationship between valence and arousal was statistically significant across sounds, but manifested only for the negative, unpleasant sounds. While unpleasant sounds reliably elicited heightened arousal, pleasant sounds did not conform to a single arousal profile. Instead, they spanned a broader spectrum, suggesting that pleasant auditory stimuli can be either soothing or stimulating depending on their specific characteristics. This deviates from many observations in emotional research as the valence/arousal “affective space” often resembles a boomerang-shaped distribution – characterized by heightened arousal at both ends of the valence spectrum and lower arousal for the more neutral stimuli [21–24]. On the other hand, our overall correlation findings are consistent with those of Hocking and collaborators [12], who developed the NESSTI databaset of environmental sounds and also reported a negative correlation between emotional valence and arousal. The inconsistent characterization of the shape of the affective space across studies may be explained by differences in the stimulus categories included in the databases used across these studies, and specifically by the lack of high-valence, high-arousal stimuli in the NESSTI and in the Marcell database used in the present study. By contrast, databases that include stimuli with erotic content, such as the IADS-2 [11], the extended IADS (IADS-E: Yang, Makita [23]), the International Affective Picture System (IAPS: Lang, Bradley [25]), and the Open Affective Standardized Image Set (OASIS: Kurdi, Lozano [22]) have all yielded boomerang-shaped distributions of ratings [22–24]. It is worth noting that the shape of the distribution does not appear to depend on the broad ‘person’ category (which included erotic content) in Kurdi et al. (2017 [22]; see their Fig 5). A boomerang-shaped distribution was also observed for vocal bursts created from recordings from the Montreal Affective Voices [26], with vocal expressions of pleasure serving as the high-valence, high-arousal category [21]. These findings highlight the importance of stimulus diversity in shaping the affective space, and caution against overgeneralizing from databases with limited emotional range. Although the Marcell database lacks high-valence high-arousal sounds, it remains a valuable resource to offer insights into how individuals emotionally respond to a broad range of everyday sounds. Its strength lies in capturing naturalistic auditory experiences that are relevant across diverse contexts. However, the absence of stimuli that typically elicit strong positive high-arousal responses may limit its ability to fully capture the upper-right quadrant of the affective space. Recognizing these boundaries not only helps contextualize the current findings but also highlights the potential for future studies to expand stimulus diversity and explore how hearing status modulates affective sound perception.

### Predictors of valence and arousal

While some research has highlighted a tendency for older adults to exhibit a positivity bias in emotional processing [27,28], our findings do not support this age-related trend. The results of the current study align with those of Picou [5], who also observed no notable difference between emotional responses of younger and older adults with normal hearing. However, it should be noted that in the current study age range is limited and thus the findings should be interpreted as relating to middle-aged adults rather than a broader age range. Additionally, although participants in our study self-reported normal hearing, it is important to consider that undiagnosed age-related hearing decline may still be present to varying degrees, potentially influencing emotional processing [4,5,7]. Nonetheless, in our sample, the self-reported hearing difficulty in everyday listening situations did not emerge as a significant predictor of affective sound evaluations. This can likely be attributed to low variability in hearing abilities.

### Study considerations

Although normative data are established, it is important to consider certain methodological aspects. Data collection was conducted online, and despite the inclusion of a headphone screening test, we cannot entirely rule out the possibility that some participants used device speakers. Nevertheless, we do not consider this a major limitation. Prior research by Picou and Buono [9] demonstrated that emotional responses to environmental sounds were not significantly affected by the type of transducer (headphones vs. loudspeakers) or by moderate variations in stimulus intensity (50–65 dBA). Based on this evidence, we believe that any potential deviations in listening setup among participants likely had minimal influence on the overall findings.

While we relied on self-reported normal hearing rather than objective audiometric testing, which could introduce some bias – particularly given the age range of our sample – this approach is not without precedent. Notably, the Australian Blue Mountains Hearing Study [29] found that 82% of individuals who reported no hearing difficulties were confirmed to have normal hearing through standard audiometry. This suggests that self-report, despite its limitations, can serve as a reasonably reliable proxy for hearing status in studies where formal testing is not feasible.

### Conclusion

This study established normative valence and arousal ratings for environmental sounds in middle-aged to older adults with self-reported normal hearing, capturing a broad emotional range. There was a moderately strong negative correlation between valence and arousal: participants generally experienced unpleasant sounds as more arousing, while pleasant sounds varied in arousal. This pattern supports the idea that valence and arousal are interrelated, rather than independent emotional dimensions. Further, age, gender, hearing-related lifestyle, hearing demands, and self-reported hearing difficulties did not significantly predict responses. These findings suggest that within a group of middle-aged to older adults with normal hearing, such factors may not strongly shape emotional reactions to environmental sounds. The normative data provided in this study can serve as a reference for the hearing research community when analyzing emotional responses in various middle-aged to older adult populations.

## Supporting information

S1 Table Mean valence and arousal ratings for individual sound tokens. The data are identical to those reported in Table 1 of the manuscript, but are provided here in Excel format to facilitate further analysis by interested readers.
(XLSX)

## Abbreviations

HEARLI-Q: Hearing-Related Lifestyle Questionnaire
HP: Huggins pitch
IADS: International Affective Digitized Sounds corpus
IAPS: International Affective Picture System corpus
NESSTI: Norms for Environmental Sound Stimuli
OASIS: Open Affective Standardized Image Set
SAM: Self-Assessment Manikin
SD: Standard deviation.
